# Uptake of the proteins HTRA1 and HTRA2 by cells mediated by calcium phosphate nanoparticles

**DOI:** 10.3762/bjnano.8.40

**Published:** 2017-02-07

**Authors:** Olga Rotan, Katharina N Severin, Simon Pöpsel, Alexander Peetsch, Melisa Merdanovic, Michael Ehrmann, Matthias Epple

**Affiliations:** 1 Inorganic Chemistry and Center for Nanointegration Duisburg-Essen (CeNIDE), University of Duisburg-Essen, Universitaetsstr. 5-7, D-45117 Essen, Germany,; 2 Centre for Medical Biotechnology, Faculty of Biology, University of Duisburg-Essen, Universitaetsstr. 5-7, D-45117 Essen, Germany

**Keywords:** calcium phosphate, endocytosis, nanoparticles, proteins

## Abstract

The efficient intracellular delivery of (bio)molecules into living cells remains a challenge in biomedicine. Many biomolecules and synthetic drugs are not able to cross the cell membrane, which is a problem if an intracellular mode of action is desired, for example, with a nuclear receptor. Calcium phosphate nanoparticles can serve as carriers for small and large biomolecules as well as for synthetic compounds. The nanoparticles were prepared and colloidally stabilized with either polyethyleneimine (PEI; cationic nanoparticles) or carboxymethyl cellulose (CMC; anionic nanoparticles) and loaded with defined amounts of the fluorescently labelled proteins HTRA1, HTRA2, and BSA. The nanoparticles were purified by ultracentrifugation and characterized by dynamic light scattering and scanning electron microscopy. Various cell types (HeLa, MG-63, THP-1, and hMSC) were incubated with fluorescently labelled proteins alone or with protein-loaded cationic and anionic nanoparticles. The cellular uptake was followed by light and fluorescence microscopy, confocal laser scanning microscopy (CLSM), and flow cytometry. All proteins were readily transported into the cells by cationic calcium phosphate nanoparticles. Notably, only HTRA1 was able to penetrate the cell membrane of MG-63 cells in dissolved form. However, the application of endocytosis inhibitors revealed that the uptake pathway was different for dissolved HTRA1 and HTRA1-loaded nanoparticles.

## Introduction

Many receptors for drugs or proteins are located inside cells [1–2]. However, because many biomolecules are not able to penetrate the cell membrane on their own, a suitable carrier is required [3–4]. Nanoparticles are readily taken up by cells via endocytosis and are easily able to deliver their cargo into cells across the cell membrane [5–7]. Calcium phosphate nanoparticles have demonstrated to be very efficient to transport (bio)molecules into cells [8–9]. For instance, nucleic acids like DNA [10–13], siRNA [14–17] and µRNA [18] have been successfully introduced to perform transfection, gene silencing, prophylactic and therapeutic vaccination [19–21]. All kinds of synthetic molecules and biomolecules can be transported across the cell membrane with the help of calcium phosphate nanoparticles [8]. The presence of calcium phosphate in human hard tissue renders them inherently biocompatible [22] unless the nanoparticle dose is very high when it may cause an intracellular disturbance of the calcium level [23–27]. After cellular uptake, calcium phosphate nanoparticles are dissolved in the acidified lysosomes and finally excreted in ionic form [28].

The high temperature requirement A (HtrA) family of serine proteases belongs to the core set of proteases found in cells and is widely conserved in single and multicellular organisms [29]. HtrAs are unique serine proteases. Besides their trypsin-like protease domain, they possess at least one C-terminal PDZ domain and can form higher oligomers [30]. The main functions of its family members are key aspects of the protein quality control process [29]. The best-studied members of this protein family in humans are HTRA1 and HTRA2 that are both involved in tumour suppression and in the control of proliferation, migration, and neurodegeneration [31]. The *HTRA1* gene (previously termed *PRSS11*) was initially identified in human fibroblasts [32].

Numerous experimental findings suggest a tumour suppressor function for HTRA1. In various cancer types, an epigenetic silencing of *HTRA1* has been observed. Moreover, a decreased *HTRA1* expression is correlated both with a reduced response to chemotherapeutics and an augmented cell migration. Additionally, a reduced proliferation of tumour cells and a decreased tumour growth have been observed upon *HTRA1* overexpression [33–34].

A wide range of extracellular matrix proteins has been reported as substrates of the protease HTRA1. Among them are fibronectin, decorin, fibromodulin, aggrecan, type ii collagen, biglycan, clusterin, ADAM9, vitronectin, α-2-macroglobulin, and Aβ, a fragment of the amyloid precursor protein [35–38]. The degradation of extracellular matrix components and its strong upregulation in patient samples indicates the involvement of HTRA1 in arthritic diseases where it may affect the degradation of cartilage as well as inflammation [38]. These and other reports suggest that HTRA1 has at least two cellular locations. While most of the produced HTRA1 is secreted into the extracellular space [37,39], about 20% are cytoplasmic and predominantly attached to microtubules and the plasma membrane. It is unknown, however, how its cellular distribution is regulated.

HTRA2 is a human serine protease located in the intermembrane compartment of mitochondria [40]. It is known to be involved in mitochondrial quality control, namely through interactions with the antiapoptotic protein HAX-1 [41]. The degradation of this protein by HTRA2 induces autophagy, resulting in the clearance of damaged mitochondria.

Similar as for HTRA1, the functional unit of HTRA2 is a trimer. Each protomer contains a trypsin-like protease domain and one C-terminal PDZ domain. The proteolytic activity can be modulated by binding of the PDZ domain to the C-terminus of other proteins. For a balanced mitochondrial homeostasis, a normal HTRA2 function is necessary in mice and humans [42]. It has been shown that mice with the inactivated form of HTRA2 (mutation of the active site) display phenotypes similar to Parkinson's disease [43]. Neurodegenerative disorders are strongly associated with the aggregation of proteins or protein fragments as well as the accumulation of unfolded proteins in mitochondria [36]. This implies that the HTRA2 protease plays a significant role in maintaining cellular homeostasis by means of protein quality control. The spontaneous uptake of recombinantly purified HTRA1 by HEK 293T cells has been used as an experimental tool [44], but the uptake of HTRA2 has not been reported so far.

Here, we have addressed the question whether the large functional proteins HTRA1 and HTRA2 can penetrate the cell membrane on their own, whether this depends on the cell type, which endocytic pathways might be employed, and whether calcium phosphate nanoparticles can act as carriers if a protein should not be able to penetrate the cell membrane by itself.

## Materials and Methods

### Production, purification, and fluorescence labelling of the proteins HTRA1 and HTRA2

HTRA1 was produced and purified as described previously [45]. BL21 DE3-Rosetta *E. coli* cells were used to express HTRA2 with an N-terminal His-tag (pET28a Vector containing codons 134-458 of HTRA2, a kind gift from Antonis S. Zervos, University of Central Florida). HTRA2 expression was induced with 500 µM IPTG at 16 °C for 16 h. The cells were harvested by centrifugation at 6,000*g* for 10 min at 4 °C and lysed at 12 bar with two passes in a microfluidizer in 20 mL ice-cold lysis buffer (50 mM NaH_2_PO_4_, pH 8.0, 300 mM NaCl) per 1 L bacterial culture. Lysates were cleared by centrifugation at 30,000*g* for 30 min at 4 °C. The supernatant was added to 5 Ni-TED 2,000 columns (Macherey-Nagel) per 1 L of expression culture that had been equilibrated with 40 column volumes of lysis buffer. The columns were then washed with 15 column volumes of lysis buffer, followed by wash buffer I (50 mM NaH_2_PO_4_, pH 8.0, 1 M NaCl) and wash buffer II (50 mM NaH_2_PO_4_, pH 8.0, 200 mM NaCl, 20 mM imidazole). HTRA2 was eluted with 3 column volumes of elution buffer I (50 mM NaH_2_PO_4_, pH 8.0, 300 mM NaCl, 250 mM imidazole) and elution buffer II (50 mM NaH_2_PO_4_, pH 8.0, 300 mM NaCl, 500 mM imidazole). Fractions containing HTRA2 were pooled and concentrated with protein concentrator spin columns (Vivaspin 20 MWCO 50,000, GE Healthcare Life Sciences). The concentrated protein was diluted with lysis buffer to a final imidazole concentration of 20 mM. The sample was then added to 2 mL Ni-NTA superflow resin (QIAGEN) per 1 L of expression culture that had been equilibrated with 40 column volumes of lysis buffer. The columns were then washed as described for the Ni-TED columns. Additionally, a third washing step with 40 column volumes of wash buffer III was included (50 mM NaH_2_PO_4_, pH 8.0, 300 mM NaCl, 70 mM imidazole). HTRA2 was then eluted with 6 column volumes of elution buffer II. Fractions containing HTRA2 were pooled, concentrated, buffer exchanged into storage buffer (50 mM Tris, pH 8.0, 150 mM NaCl, 10% glycerol), shock-frozen in liquid nitrogen, and stored at −80 °C.

The protein concentrations were determined with a Bradford assay (Roti-Nanoquant). HTRA1 and HTRA2 proteins were fluorescently labelled with the amine-reactive fluorescent dye Alexa Fluor^®^ 488 Carboxylic Acid, 2,3,5,6-tetrafluorophenyl ester (Thermo Fisher Scientific) following the manufacturer's instructions. These labelled proteins are denoted as HTRA1-488 and HTRA2-488 in the following.

Fluorescein-isothiocyanate (FITC)-labelled bovine serum albumin (denoted as BSA-FITC in the following) was obtained from Sigma-Aldrich (Germany).

### Synthesis of the protein-loaded calcium phosphate nanoparticles

The nanoparticles were synthesized as described in the following, according to synthetic procedures described previously [7,46]. Aqueous solutions of calcium nitrate (6.25 mM; Merck p.a.) and diammonium hydrogen phosphate (3.74 mM; Merck p.a.) were rapidly mixed by pumping them into a glass vessel with a peristaltic pump. The pH of both solutions was adjusted beforehand with aqueous NaOH (0.1 M; Merck, p.a.) to pH 9. A few seconds after mixing, 1 mL of the calcium phosphate nanoparticle dispersion was taken with a syringe and mixed with 0.05 mL of either aqueous carboxymethyl cellulose solution (CMC; Sigma-Aldrich, *M*_w_ = 90 kDa, degree of substitution 0.7; 2 mg mL^−1^) or aqueous polyethyleneimine solution (PEI; Sigma-Aldrich, *M*_w_ = 25 kDa; 2 mg mL^−1^), respectively, to achieve the colloidal stability of the nanoparticle dispersion. These particles were used either as-prepared (denoted as CaP/CMC and CaP/PEI) or further loaded with proteins.

For loading with proteins, 0.5 mL of one dissolved fluorescently labelled protein (HTRA1-488: 1 mg mL^−1^; BSA-FITC: 1 mg mL^−1^; HTRA2-488 0.7 mg mL^−1^) was added to the dispersion under thorough stirring. All particles were separated from dissolved counterions and nonadsorbed molecules by ultracentrifugation (21,000*g*, 30 min) with subsequent redispersion in the same volume of water with a sonotrode (cycle 0.8, amplitude 60%, 2 min). All inorganic salts were of p.a. quality. Ultrapure water (Purelab ultra instrument from ELGA) was used for all preparations. All formulations were prepared and analysed at room temperature.

### Analytical methods

Dynamic light scattering (DLS) and zeta potential determinations were performed with a Zetasizer Nano series instrument (Malvern Nano-ZS, laser: λ = 532 nm) using the Smoluchowski approximation and taking the data from the Malvern software without further correction. The particle size data refer to scattering intensity distributions (*z*-average). Scanning electron microscopy (SEM) was performed with an ESEM Quanta 400 instrument with gold/palladium-sputtered samples. Centrifugation was performed at 4 °C with a Heraeus Fresco 21 centrifuge. The amount of calcium was determined by atomic absorption spectroscopy (AAS) with an M-Series AA spectrometer (Thermo Electron Corporation, Schwerte). The amount of fluorescently labelled protein on the nanoparticles was determined by quantitative UV spectroscopy, using previously measured calibration curves at λ = 496 nm for BSA-FITC and λ = 498 nm for HTRA1-488 and HTRA2-488 labelled proteins.

The number of particles per volume and the number of proteins per nanoparticle were computed by taking the mass of calcium phosphate in the dispersion and assuming spherical nanoparticles with the diameter obtained by SEM.

[1]
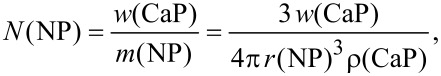


where *N*(NP) is the number of nanoparticles per m^3^, *w*(CaP) the mass concentration of calcium phosphate in kg m^−3^, *r*(NP) the average radius of one nanoparticle (from SEM), *m*(NP) the mass of one nanoparticle, and ρ(CaP) the density of calcium phosphate, assumed as hydroxyapatite (Ca_5_(PO_4_)_3_OH; 3,140 kg m^−3^). The following values were used for the computation: *w*(Ca^2+^) = 0.0148 kg m^−3^ for CaP/CMC/HTRA1 and 0.0099 kg m^−3^ for CaP/PEI/HTRA1; *c*(Ca_5_(PO_4_)_3_OH) = 0.037 kg m^−3^ for CaP/CMC/HTRA1 and 0.025 kg m^−3^ for CaP/PEI/HTRA1; *r*(NP) = 38.5 nm for CaP/CMC/HTRA1 and 18.5 nm for CaP/PEI/HTRA1; *V*(NP) = 2.4·10^−22^ m^3^ for CaP/CMC/HTRA1 and 2.7·10^−23^ m^3^ for CaP/PEI/HTRA1; *m*(NP) = 7.5·10^−19^ kg for CaP/CMC/HTRA1 and 8.3·10^−20^ kg for CaP/PEI/HTRA1.

The concentration of molecules on the nanoparticles was calculated by subtraction of the protein concentration in the supernatant by quantitative UV–vis spectroscopy using a calibration curve from the original concentration of the molecule in the nanoparticle dispersion. This is equivalent to the protein concentration on the nanoparticles. The number concentration of fluorescent molecules can then be calculated as follows:

[2]
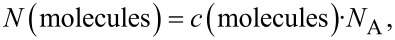


where *N*(molecules) is the number of fluorescent molecules per m^3^, *c*(molecules) the molar concentration of the fluorescent molecules in the nanoparticle dispersion, and *N*_A_ is Avogadro’s number (6.022·10^23^ mol^−1^).

The number of molecules per nanoparticle was obtained by dividing the number of molecules per m^3^ by the number of nanoparticles per m^3^. The number of functionalized nanoparticles per cell in the cell culture experiments was calculated accordingly.

### Cell line culture and imaging

Human epithelial cervical cancer cells (HeLa) and human osteosarcoma cells (MG-63) cell lines were cultured in DMEM, supplemented with 10% fetal calf serum (FCS) under 37 °C, 5% CO_2_ and humidified atmosphere and cultivated according to standard cell culture protocols. A primary cell culture of human mesenchymal stem cells (hMSCs) was cultivated using mesenchymal stem cell (MSC) growth medium, supplemented according to the standard cultivation protocol. Approximately 12 h prior to the experiments, the cells were trypsinized and seeded in 24-well cell culture plates with 1·10^5^ cells per well in 0.5 mL cell medium.

The incubation with nanoparticles was carried out by adding a 50 µL aliquot of the particle dispersion to each well (i.e., the nanoparticle dispersion was diluted 1:11). The cells were incubated for 3 h (unless indicated otherwise). Subsequently, the cell culture medium was removed. The cells were washed three times with phosphate-buffered saline (PBS). Therefore, only the nanoparticles that either had been taken up by the cells or were strongly adsorbed on the cellular surface remained.

In order to obtain adherent cells, THP-1 cells (a human acute monocytic leukemia cell line) were first seeded in 24-well plates (Sarstedt, USA) at a density of 5·10^5^ cells per well in 0.5 mL of cell medium, then differentiated into macrophages by the addition of 100 nM of PMA (4β-phorbol-12β-myristate-13α-acetate, Sigma-Aldrich, USA) solution per well, and finally incubated for three days. Afterwards, the cell medium was changed, and the cells were then treated like the other cell lines.

Light and fluorescence microscopy were performed on a Zeiss Axiovert 40 CFL instrument (Carl Zeiss, Goettingen, Germany) and a Keyence Biorevo BZ-9000 instrument (Osaka, Japan), both equipped with filters for tetramethylrhodamine (TRITC) (excitation: 540 nm, emission: 605 nm), green fluorescent protein (GFP) BP (excitation: 470 nm, emission: 535 nm) and 4',6-diamidino-2-phenylindole (DAPI) (excitation: 360 nm, emission: 460 nm) channels. Confocal laser scanning microscopy (CLSM) was performed on a Leica SP5 confocal inverse CLSM. The cells were stained with DAPI (nucleus) and Cell mask™ (cell membrane) to indicate the cellular uptake of the nanoparticles. To elucidate the uptake mechanism, several endocytosis inhibitors were used as described in Table 1.

**Table 1:** Endocytosis inhibitors used in the cell culture experiments.

Inhibitor	Pathway/protein affected	*c*(inhibitor) in stock solution [µg mL^−1^]	*V*(inhibitor) added to each well [µL]	*c*(inhibitor) in each well

Wortmannin	Macropinocytosis	10	20	100 ng mL^−1^
LY294002	Macropinocytosis	1,000	40	20 µg mL^−1^
Nystatin	Caveolin-mediated endocytosis/lipid rafts	2,000	10	10 µg mL^−1^
Nocodazole	Microtubules of the cytoskeleton	1,000	20	10 µg mL^−1^
Chlorpromazine	Clathrin-mediated endocytosis	100	20	1 µg mL^−1^

Inhibitor concentrations and application procedures were carried out as reported previously [47]. Briefly, one day before the experiment, MG-63 cells were seeded in 6-well plates with 1·10^6^ cells per well in 2 mL of cell culture medium. On the following day, the cell culture medium was replaced by fresh medium with or without the inhibitors to a total volume of 2 mL and incubated for 30 min. Moreover, the cells were cultivated either at 37 °C (positive control) or at 4 °C (negative control for endocytosis). Subsequently, the nanoparticles loaded with protein or the protein alone were added to the corresponding cell samples and incubated for another 3 h. The cells were then harvested for flow cytometry analysis as follows. All samples were washed three times with PBS, detached with trypsin/EDTA (3 min at 37 °C) and transferred into 15 mL Falcon tubes by adding 3 mL of the cell culture medium to each cell sample. The cells were then washed three times by centrifugation (1,700*g*, 5 min, 4 °C) and subsequently redispersed in 4 mL PBS. After the final centrifugation, all samples were redispersed in 2% formaldehyde solution and incubated for 20 min at room temperature in the dark. Then 0.6 mL of PBS were added to each sample and the cells were again washed three times by centrifugation (1,700*g*, 5 min, 4 °C) and subsequently redispersed in 3 mL PBS. After the last washing step, each sample was redispersed in 1 mL PBS and stored in the dark at 4 °C. The prepared cell samples were then transferred to flow cytometry tubes for measurements.

### Flow cytometry analysis

Flow cytometry analysis was performed using a BD FACSCalibur™ instrument (BD Biosciences) and analysis was done using the FlowJo analysis software. Side-scatter height versus side-scatter width (SSC-H versus SSC-W) and forward-scatter height versus forward-scatter width (FSC-H versus FSC-W) were used to gate out cell aggregates and to ensure single cell analysis. 10,000 cells were analysed per sample, and all samples were measured in duplicates. The uptake of fluorescently labelled proteins was determined by measuring the fluorescence intensity of single cells using the FITC channel.

## Results and Discussion

**Figure 1:**
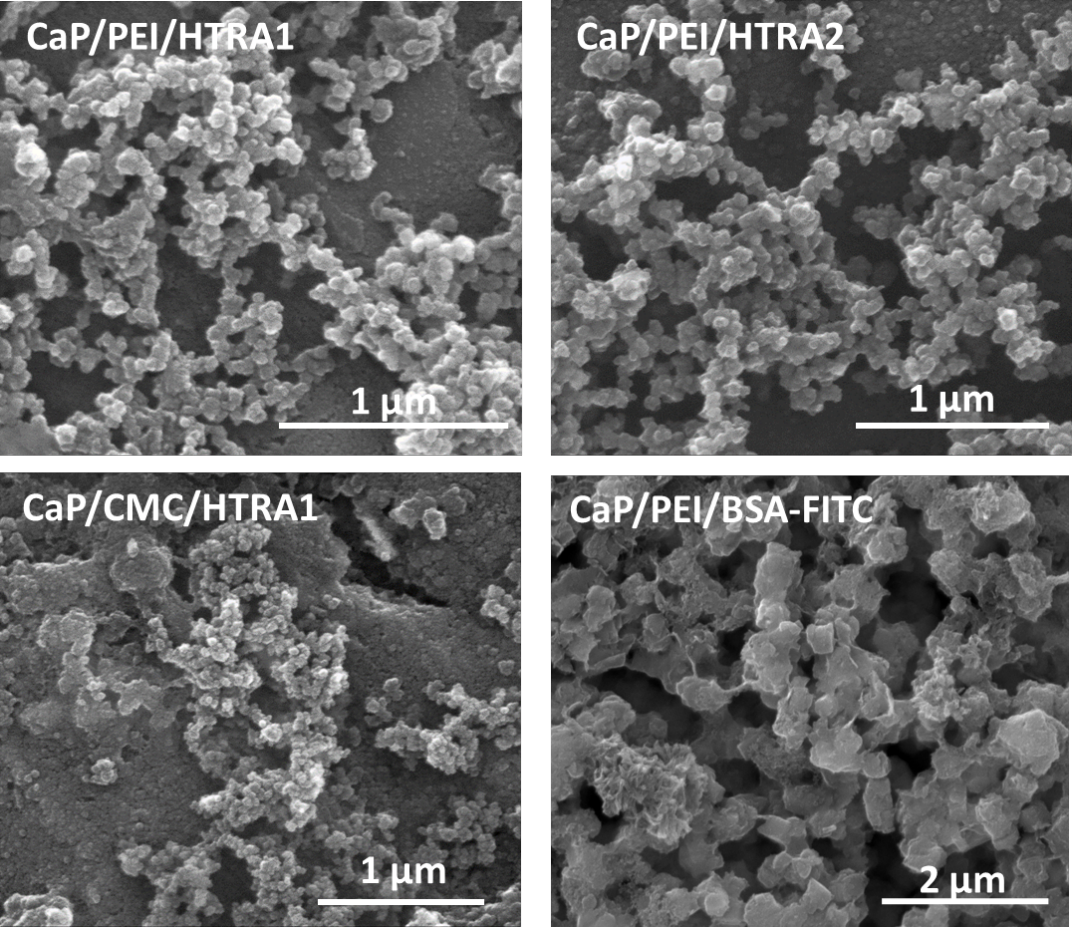
Representative scanning electron micrographs of purified, functionalized calcium phosphate nanoparticles.

To investigate whether calcium phosphate nanoparticles are suitable mediators for the uptake of HTRA1 and HTRA2 by human cells, calcium phosphate nanoparticles were loaded with either HTRA1, HTRA2, or BSA. BSA served as a nonfunctional control protein. These protein-functionalized nanoparticles had an almost spherical morphology and a diameter between 30 and 150 nm as shown by SEM (Figure 1).

Initially, the functionalized nanoparticles were physicochemically characterized (Table 2). All nanoparticles had a hydrodynamic diameter between 250 and 350 nm. The zeta potential reflects the corresponding charge of the polyelectrolyte polymer used for the stabilization (CMC: negative charge, PEI: positive charge) that indicates the colloidal stability of the nanoparticles in dispersion. Both cationic (with PEI) and anionic (with CMC) nanoparticles were prepared. Note that the difference between the particle size by SEM and by dynamic light scattering (DLS) indicates a moderate degree of agglomeration of the nanoparticles in pure water. It can be safely assumed that the dispersion in biological media containing proteins (like RPMI/10% FCS) is much better due to the formation of a protein corona [48–49].

**Table 2:** Characterization of protein-loaded nanoparticles by dynamic light scattering (DLS) and UV–vis spectroscopy (all data recorded in pure water). The particles functionalized with carboxymethylcellulose (CMC) were anionic and the particles functionalized with polyethyleneimine (PEI) were cationic. The concentration of calcium phosphate was computed from the calcium concentration as described in the experimental section. All concentrations refer to the nanoparticle stock solutions before the addition to the cell culture well and the subsequent dilution. The number of protein molecules per nanoparticle is based on the particle diameter from scanning electron microscopy (see Table 3). PDI: polydispersity index, from DLS.

Protein	Sample	Diameter, DLS [nm]	PDI, DLS	Zeta potential, DLS [mV]	*c*(protein) [µg mL^−1^]	*c*(calcium phosphate) [µg mL^−1^]	*N*(protein) per nanoparticle

HTRA1	CaP/CMC/HTRA1	247	0.321	−16	168	37.1	5.6·10^4^
	CaP/PEI/HTRA1	292	0.325	+18	410	24.8	2.3·10^4^
HTRA2	CaP/CMC/HTRA2	696	0.565	−16	255	3.0	4.7·10^5^
	CaP/PEI/HTRA2	450	0.369	+17	226	6.2	2.6·10^5^
BSA	CaP/CMC/BSA-FITC	345	0.395	−22	165	25.0	2.1·10^4^
	CaP/PEI/BSA-FITC	975	0.707	+14	229	24.8	2.9·10^4^

The concentration of the labelled proteins was determined by UV–vis spectroscopy (Table 2). Table 3 gives the quantification data of the functionalized nanoparticles, where the amount of protein per nanoparticle and per cell is also given. Clearly, the number of nanoparticles and the number of protein molecules is much higher than the number of cells in a well. An interesting fact is the high loading of nanoparticles with proteins. This results has been experimentally verified a number of times by us. Due to the multiple purification steps (centrifugation and redispersion), the presence of free (dissolved) protein can be excluded. We can conclude that the nanoparticles contain a high amount of adsorbed protein in all cases.

**Table 3:** Computed number of protein molecules per nanoparticle (NP) and per cell (for 1·10^5^ cells in 0.5 mL cell medium + 0.05 mL NP dispersion). These numbers apply to all cell types used, e.g., HeLa, MG-63 and THP-1 cells. All concentrations refer to the state after 1:11 dilution in the cell culture well.

Sample	Diameter (NP), SEM [nm]	*n*(protein) [mol per well]	*N*(protein) [per well]	*N*(NP) [per well]	*N*(protein) [per cell]

CaP/CMC/HTRA1	77	2.3·10^−10^	1.4·10^14^	2.5·10^9^	1.4·10^9^
CaP/PEI/HTRA1	37	5.6·10^−10^	3.4·10^14^	1.5·10^10^	3.4·10^9^
CaP/CMC/HTRA2	60	3.3·10^−10^	1.9·10^14^	4.2·10^8^	1.9·10^9^
CaP/PEI/HTRA2	65	2.9·10^−10^	1.8·10^14^	6.9·10^8^	1.8·10^9^
CaP/CMC/BSA-FITC	60	1.2·10^−10^	7.5·10^13^	3.5·10^9^	7.5·10^8^
CaP/PEI/BSA-FITC	60	1.7·10^−10^	1.0·10^14^	3.5·10^9^	1.0·10^9^

**Figure 2:**
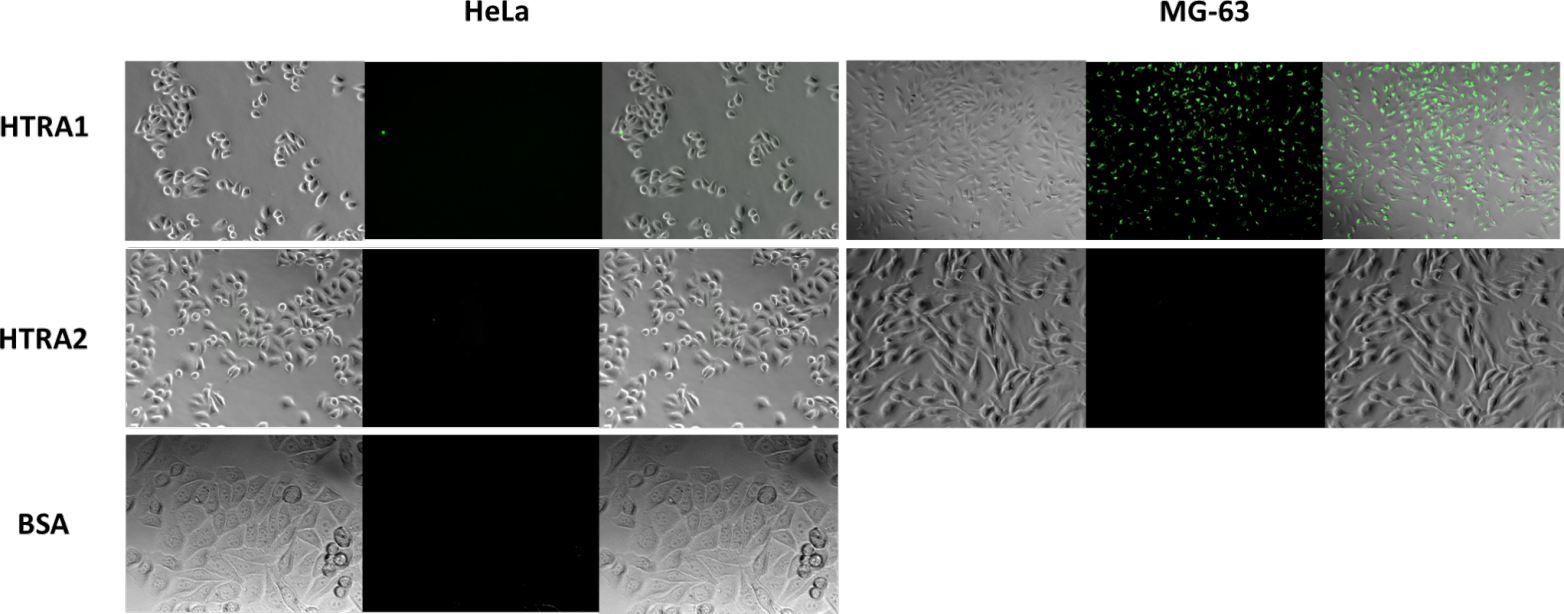
Uptake of the dissolved fluorescently labelled proteins HTRA1-488, HTRA2-488, and BSA-FITC by HeLa and MG-63 cells. The only significant uptake was observed for HTRA1-488 by MG-63 cells. In each image set: left: Light microscopy, centre: fluorescence microscopy (labelled proteins on nanoparticles), right: overlay.

First, we measured the uptake of the fluorescently labelled proteins HTRA1-488, HTRA2-488 and BSA-FITC alone by two cell lines: HeLa and MG-63. The cells were incubated with the dissolved proteins for 3 h at 168 µg mL^−1^ for HTRA1-488, 255 µg mL^−1^ for HTRA2-488 and 229 µg mL^−1^ for BSA-FITC. Subsequently, the cells were washed three times with PBS to remove adherent protein and then analysed with light and florescence microscopy (Figure 2). The only case where a dissolved protein was taken up by cells was for HTRA1 with MG-63 cells. In all other cases, no significant uptake of the dissolved proteins was observed.

**Figure 3:**
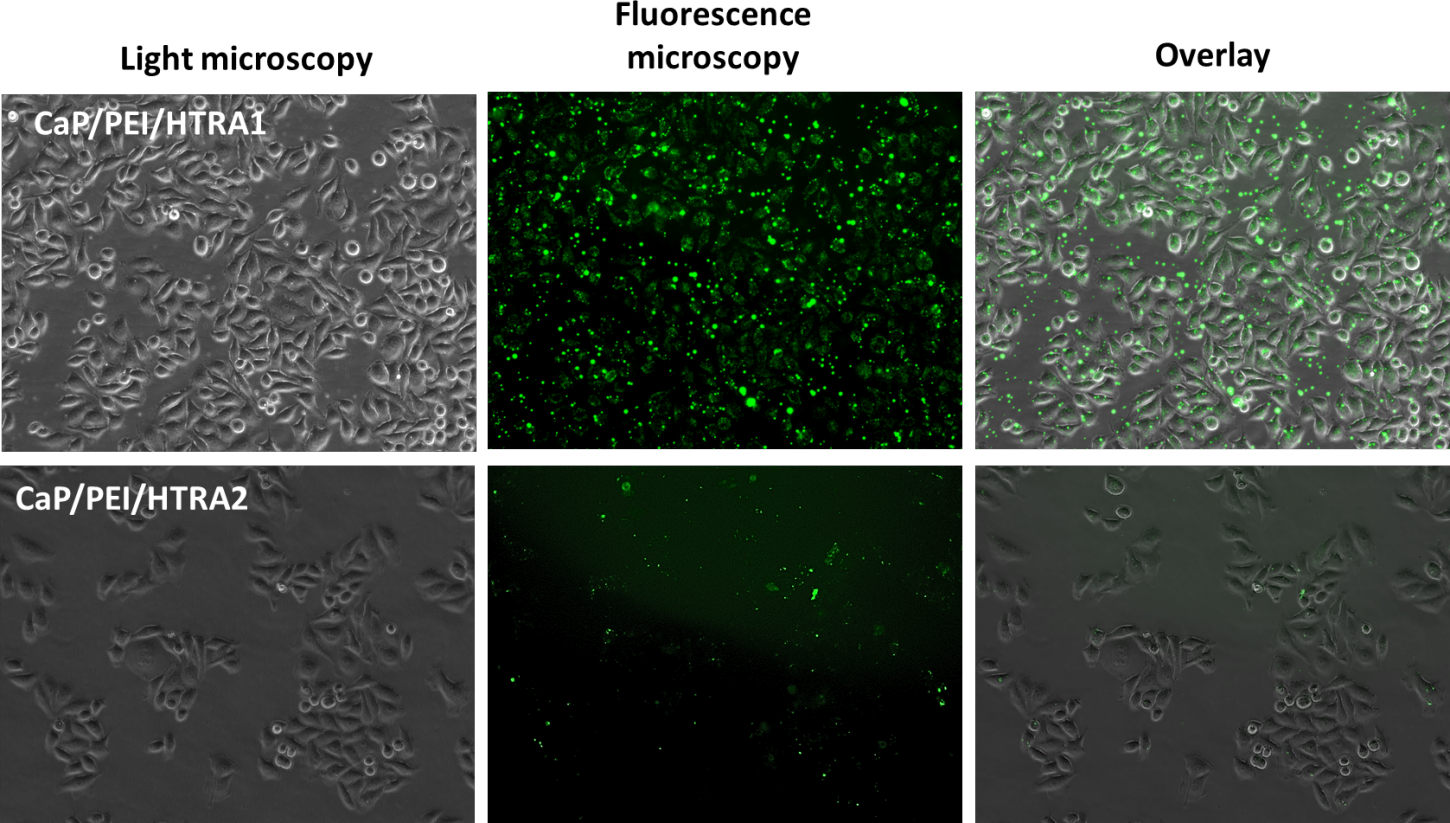
Transport of the fluorescently labelled proteins HTRA1-488 and HTRA2-488 with the help of cationic CaP/PEI/HTRA1 or CaP/PEI/HTRA2 nanoparticles into HeLa cells.

**Figure 4:**
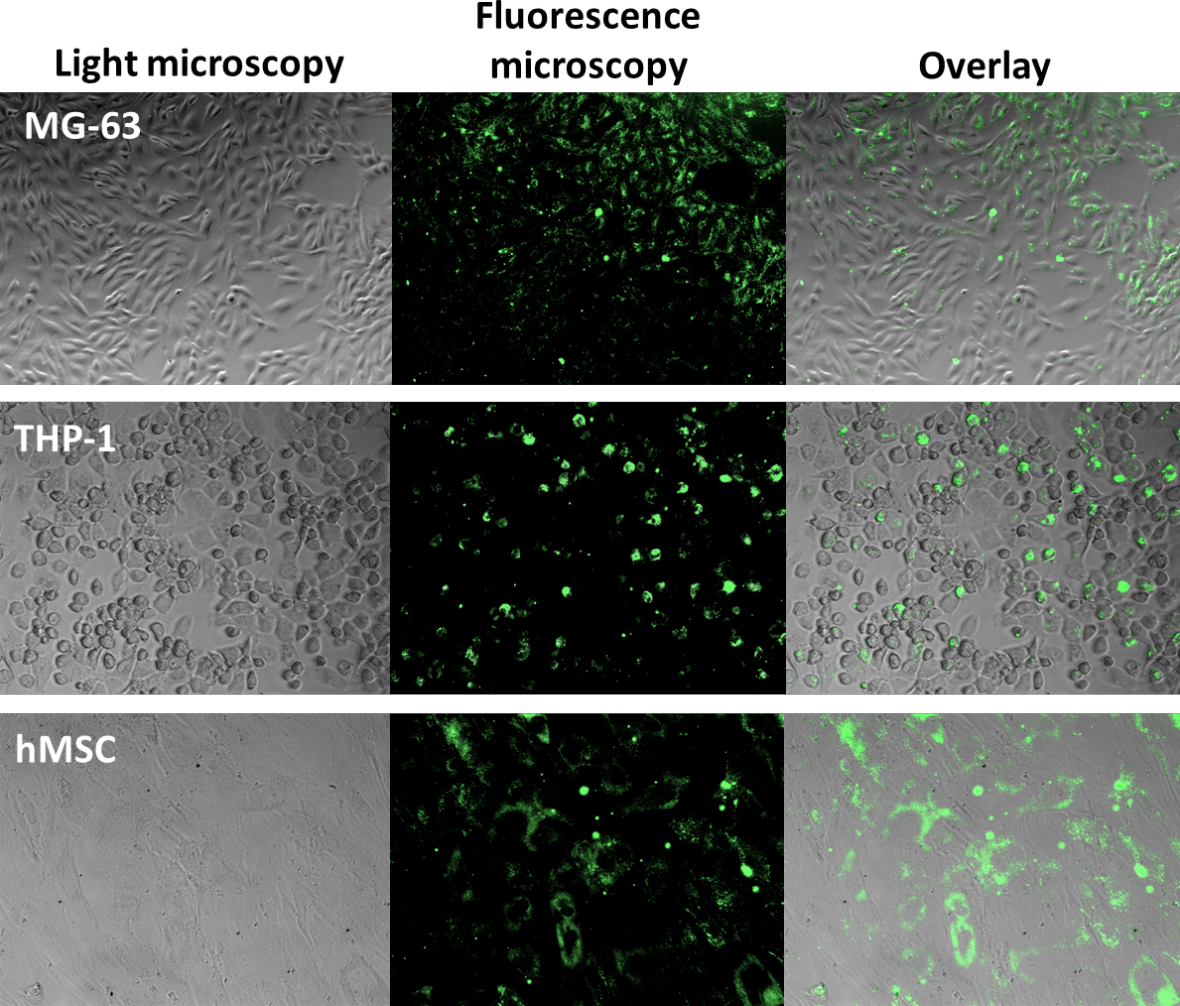
Uptake of anionic CaP/CMC/HTRA1-488 nanoparticles by different cell lines. The uptake by HeLa cells was very low (data not shown).

To deliver the proteins HTRA1-488 and HTRA2-488 to HeLa cells, we prepared cationic PEI-functionalized nanoparticles and loaded them with either HTRA1-488 or HTRA2-488 (Table 2 and Figure 3). For comparison, we prepared negatively charged CaP/CMC/HTRA1 nanoparticles. They were taken up by MG-63, THP-1 and hMSC, but HeLa cells showed only a very low uptake of these anionic nanoparticles (Figure 4). Table 4 summarizes all results of the uptake experiments, including those with cationic CaP/PEI and anionic CaP/CMC nanoparticles (i.e., without proteins).

**Table 4:** Summary of the uptake of proteins alone and proteins loaded onto cationic and anionic nanoparticles, as well as nanoparticles alone, by HeLa and MG-63 cells. Cationic nanoparticles (NPs) without proteins consisted of calcium phosphate/polyethyleneimine (CaP/PEI). Anionic nanoparticles without proteins consisted of calcium phosphate/carboxymethylcellulose (CaP/CMC). +: strong take-up by cells; 0: moderate take-up by cells; −: no uptake by cells; n.d.: not determined.

Cell line	HTRA1	HTRA2	BSA	HTRA1		HTRA2		BSA		Cationic NPs alone	Anionic NPs alone
				Cationic NPs	Anionic NPs	Cationic NPs	Anionic NPs	Cationic NPs	Anionic NPs		

HeLa	−	−	−	+	0	+	−	+	0	+	+
MG-63	+	−	−	n.d.	+	+	−	+	−	n.d.	+

It has been frequently reported that positively charged nanoparticles are usually taken up better and faster by cells because of the electrostatic interaction of the negatively charged cell membrane and the positively charged surface of the nanoparticles [47,50–55]. This was confirmed in our experiments: The uptake of proteins with cationic nanoparticles was higher than with anionic nanoparticles.

The results of Table 4 indicate that in almost all cases, the cells did not take up the dissolved proteins, that anionic nanoparticles transported the proteins in some cases, and that cationic nanoparticles were always suitable carriers for the proteins.

**Figure 5:**
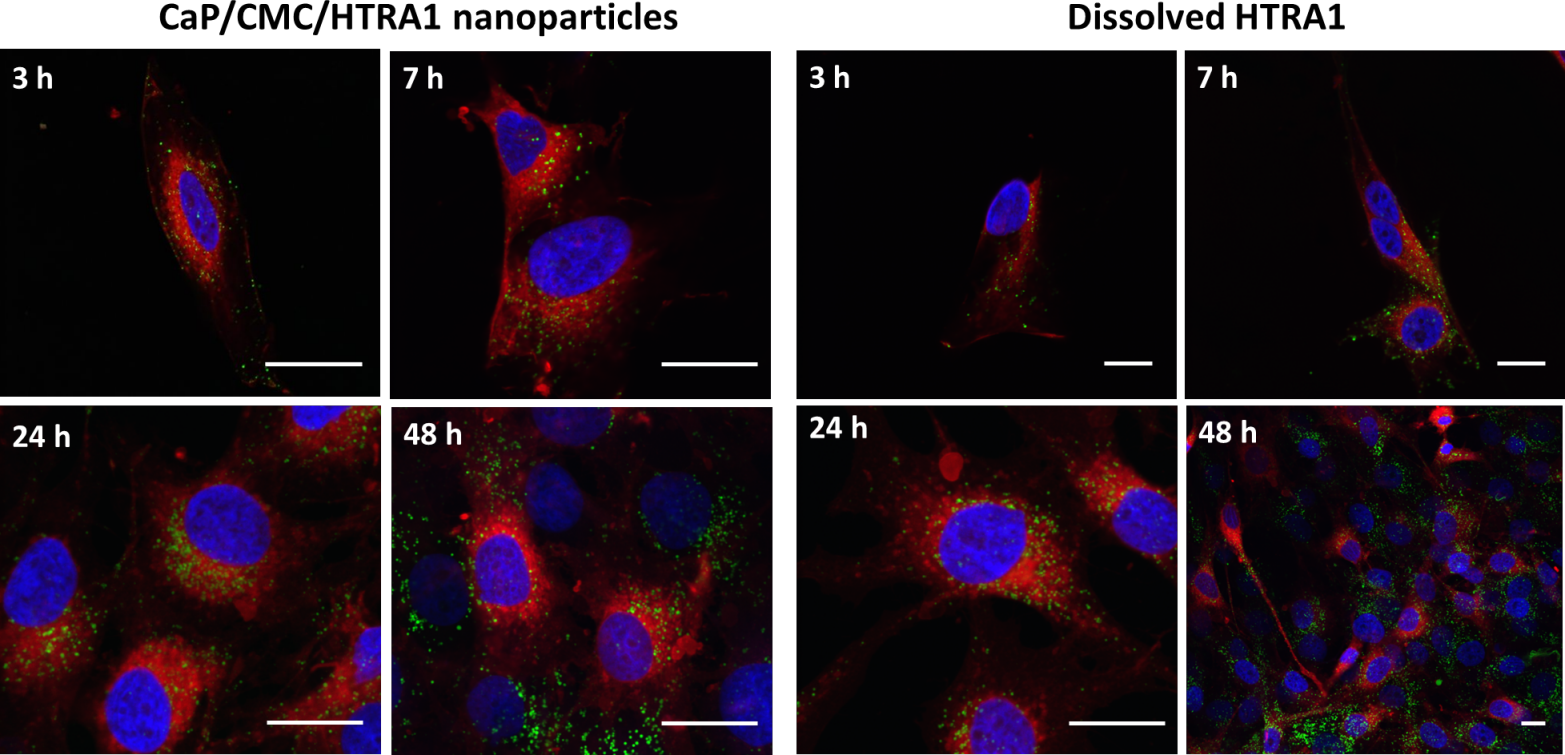
Kinetic study of the uptake of the fluorescently labelled protein HTRA1-488 (green) by MG-63 cells, both loaded onto anionic CaP/CMC nanoparticles (left) and dissolved (right). The cell nuclei were stained blue with DAPI, and the cell membrane was stained red with CellMask^TM^. Scale bar in all images: 10 µm.

It is most interesting that the MG-63 cells were able to take up HTRA1 with and without nanoparticles. It is likely, however, that the uptake mechanisms are different. Therefore, this case was studied in more detail. To compare the uptake kinetics of HTRA1 alone and loaded onto negatively charged nanoparticles, we incubated MG-63 cells with either dissolved HTRA1-488 or CaP/CMC/HTRA1-488 nanoparticles for various times, 3, 7, 24, and 48 h and followed the uptake by CLSM. For the protein alone and for the protein loaded onto nanoparticles, the amount of the internalized protein clearly increased with time (Figure 5). This indicates that MG-63 cells are constantly internalizing the HTRA1 protein, even without the help of a nanoparticle.

**Figure 6:**
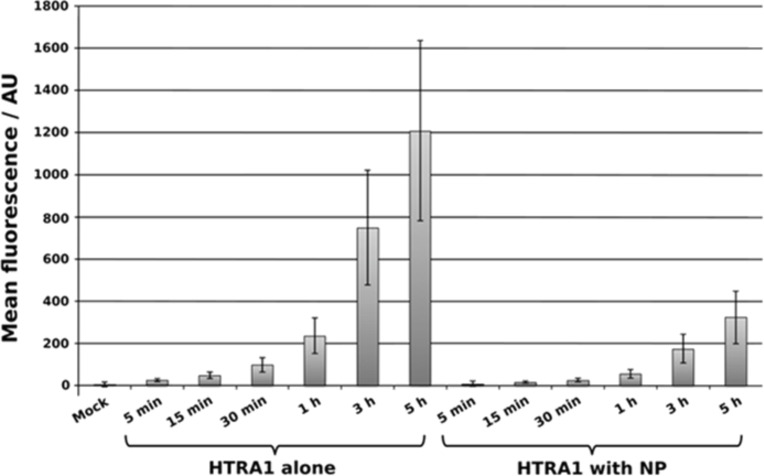
Comparative kinetic study of the uptake of CaP/CMC/HTRA1-488 nanoparticles and soluble HTRA1-488 by MG-63 cells, measured by flow cytometry.

In a parallel experiment, the protein uptake was quantified by flow cytometry. The dissolved protein was taken up much faster by MG-63 cells than the protein-loaded nanoparticles (Figure 6). An exponential increase in the fluorescence intensity was found in both cases. This suggests that HTRA1 is a highly mobile protein that is internalized probably by a different mechanism by cells in dissolved form and loaded onto nanoparticles. Despite the fact that the mean fluorescence intensity as measured by flow cytometry analysis is not fully quantitative, the high difference between HTRA1 alone and HTRA1 with nanoparticles shows that the uptake is more efficient for the dissolved protein.

**Figure 7:**
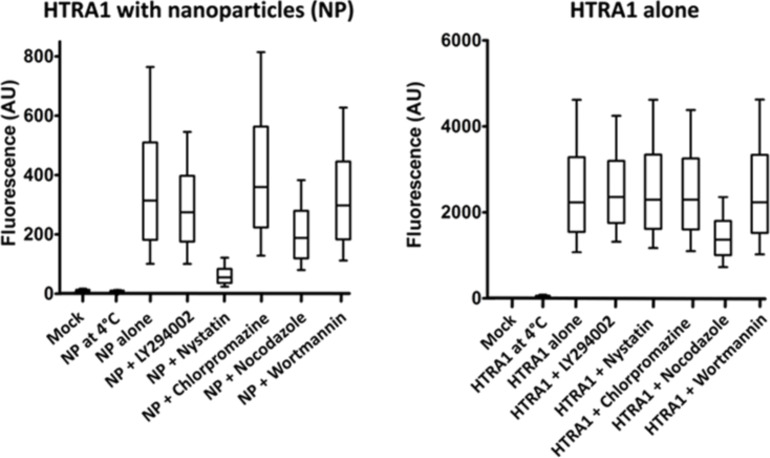
Uptake of anionic calcium phosphate nanoparticles CaP/CMC/HTRA1-488 (left) and of dissolved HTRA1-488 (right) by MG-63 cells. Different endocytotic pathways were selectively blocked with specific inhibitors. The protein uptake after 3 h incubation time was analysed by flow cytometry. Mock: Untreated cells.

The uptake mechanism was studied further by selectively blocking different endocytotic pathways in MG-63 cells with selective inhibitors (Table 1 and Figure 7).

The uptake of the CaP/CMC/HTRA1-488 nanoparticles was partially inhibited by nocodazole (which inhibits the polymerization of microtubules in the cytoskeleton) and almost completely by nystatin (which inhibits caveolin-mediated endocytosis). The uptake of the protein alone was partially inhibited only by nocodazole. This clearly shows that the uptake mechanisms are different for the dissolved protein and for protein-loaded nanoparticles. The uptake was completely inhibited at 4 °C for protein alone and nanoparticles, demonstrating the energy-dependent endocytosis in both cases.

**Figure 8:**
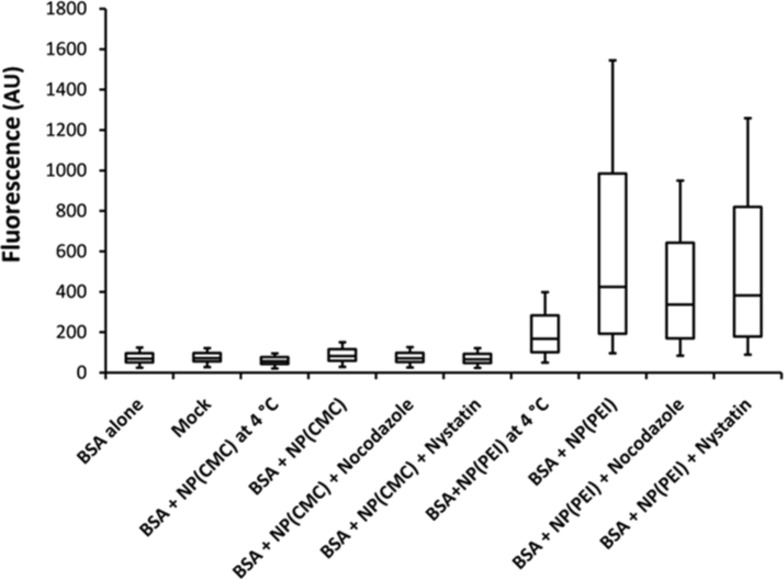
Flow cytometry analysis of the uptake of CaP/CMC/BSA-FITC and CaP/PEI/BSA-FITC nanoparticles by MG-63 cells in the presence of endocytosis inhibitors. Mock: Untreated cells.

HTRA1 is a special case of a protein that is able to penetrate the cell wall of MG-63 cells in dissolved form (Table 4). Typically, (bio)molecules do not penetrate the cell membrane in dissolved form, i.e., without the help of a nanoparticle [7–8]. The control protein BSA behaved differently. We have prepared and studied the uptake of both anionic and cationic nanoparticles loaded with BSA-FITC. Figure 8 shows that BSA-FITC alone was not taken up by MG-63 cells. Negatively charged CaP/CMC/BSA-FITC nanoparticles were taken up only to a low extent. In contrast, positively charged CaP/PEI/BSA-FITC nanoparticles were easily able to transport BSA-FITC across the cell membrane, and the transport was almost not inhibited by nocodazole and nystatin. This illustrates the importance of the nanoparticle charge for the uptake by cells.

We have previously reported that the main internalization pathway for anionic calcium phosphate nanoparticles into HeLa cells was macropinocytosis. In that case, cationic nanoparticles were also taken up much better than anionic nanoparticles [47]. This is also supported by earlier studies on the uptake of nanoparticles with different charge by cells [56–57]. It also underscores the fact that both the cell type as well as the physicochemical properties of a nanoparticle play important roles when it comes to the uptake of nanoparticles by cells [5,55,58–63]. The presence of proteins around nanoparticles may also influence their uptake [64–67]. Krais et al. have shown that serum proteins were necessary for cancer cells to take up folic acid-conjugated iron oxide nanoparticles [68]. The nature of the protein corona on the protein-loaded calcium phosphate nanoparticles after immersion in cell culture medium is unknown, but probably influenced by the particle charge [69].

## Conclusion

We have investigated the uptake of the functional proteins HTRA1 and HTRA2 by various cell lines, in dissolved form and loaded onto calcium phosphate nanoparticles. BSA was used as a control. Typically, nanoparticles are necessary as carriers for proteins across the cell membrane, with cationic nanoparticles being much more efficient than anionic nanoparticles. The surprising fact that dissolved HTRA1 was constantly taken up by MG-63 cells, even much more efficient than when loaded onto anionic nanoparticles, points to different uptake mechanisms. This was corroborated by the application of different endocytosis inhibitors that showed strong differences between dissolved HTRA1 and HTRA1 loaded onto nanoparticles. In this respect, HTRA1 appears to be a special case with a specific uptake pathway that is not used by other proteins. In all cases, calcium phosphate nanoparticles were effective transporters for proteins across the cell membrane. This is important for an efficient intracellular delivery of therapeutic proteins.
